# The relationship between spontaneous abortion and female workers in the semiconductor industry

**DOI:** 10.1186/s40557-017-0204-x

**Published:** 2017-10-09

**Authors:** Heechan Kim, Ho-Jang Kwon, Jeongbae Rhie, Sinye Lim, Yun-Dan Kang, Sang-Yong Eom, Hyungryul Lim, Jun-Pyo Myong, Sangchul Roh

**Affiliations:** 1 Department of Occupational and Environmental Medicine, Dankook University College of Medicine, 119 Dandae-ro, Dongnam-gu, Cheonan-si, Chungcheongnam-do 31116, Cheonan, Korea, Republic of; 2 Department of Preventive Medicine, Dankook University College of Medicine, Cheonan, South Korea; 3 Department of Occupational and Environmental Medicine, College of Medicine, Kyung Hee University, Seoul, Republic of Korea; 4 Department of Obstetrics and Gynecology, Dankook University College of Medicine, Cheonan, South Korea; 5 Department of Preventive Medicine and Medical Research Institute, Chungbuk National University College of Medicine, Cheongju, South Korea; 6 Department of Occupational and Environmental Medicine, College of Medicine, Seoul St. Mary’s Hospital, Catholic University of Korea, 222 Banpo-Daero, Seocho-gu, Seoul 06591, Seoul, Korea, Republic of

**Keywords:** Semiconductor industry, Occupational exposure, Spontaneous abortion, Women, Korea, Reproductive toxicity

## Abstract

**Background:**

This study investigated the relationship between job type and the risk for spontaneous abortion to assess the reproductive toxicity of female workers in the semiconductor industry.

**Methods:**

A questionnaire survey was administered to current female workers of two semiconductor manufacturing plants in Korea. We included female workers who became pregnant at least 6 months after the start of their employment with the company. The pregnancy outcomes of 2,242 female workers who experienced 4,037 pregnancies were investigated. Personnel records were used to assign the subjects to one of three groups: fabrication process workers, packaging process workers, and clerical workers. To adjust for within-person correlations between pregnancies, a generalized estimating equation was used. The logistic regression analysis was limited to the first pregnancy after joining the company to satisfy the assumption of independence among pregnancies. Moreover, we stratified the analysis by time period (pregnancy in the years prior to 2008 vs. after 2009) to reflect differences in occupational exposure based on semiconductor production periods.

**Results:**

The risk for spontaneous abortion in female semiconductor workers was not significantly higher for fabrication and packaging process workers than for clerical workers. However, when we stratified by time period, the odds ratio for spontaneous abortion was significantly higher for packaging process workers who became pregnant prior to 2008 when compared with clerical workers (odds ratio: 2.21; 95% confidence interval: 1.01–4.81).

**Conclusions:**

When examining the pregnancies of female semiconductor workers that occurred prior to 2008, packaging process workers showed a significantly higher risk for spontaneous abortions than did clerical workers. The two semiconductor production periods in our study (prior to 2008 vs. after 2009) had different automated processes, chemical exposure levels, and working environments. Thus, the conditions prior to 2008 may have increased the risk for spontaneous abortions in packaging process workers in the semiconductor industry.

**Electronic supplementary material:**

The online version of this article (10.1186/s40557-017-0204-x) contains supplementary material, which is available to authorized users.

## Background

The semiconductor industry began in the 1960s in England and the United States as a sector of the microelectronics industry; it was fully introduced in Korea during the 1970s [1]. Today, the semiconductor industry in Korea has a world-class production capacity with a high growth rate [2]. The current working population of the Korean semiconductor industry is about 107,000, accounting for 0.7% of the entire working population [3]. The percentage of female workers in this industry is 30.8%, which is higher than in other manufacturing industries [4]. In 2007, a woman working in a Korean semiconductor factory who was responsible for cleaning semiconductors died of leukemia. As a result, discussions on working environments in the semiconductor industry and occupational diseases began taking place. In 2011, the courts ruled that leukemia was related to semiconductor work. Meanwhile, 18 other workers whose jobs involved semiconductor processes were diagnosed with leukemia, aplastic anemia, lymphoma, breast cancer, and ovarian cancer; these conditions were subsequently recognized as occupational diseases by the Korea Workers’ Compensation and Welfare Service (KWCWS) and the courts. Currently, not only cancers but also diseases related to reproductive toxicity, such as infertility, are included in the list of occupational diseases in semiconductor workers by KWCWS [5]. Therefore, efforts to protect and improve the health of women in the semiconductor industry are needed.

In the semiconductor industry, the process of producing semiconductors involves the use of various chemicals and produces various by-products [6]. During this process, female workers might be exposed to chemicals that can affect their health [7]. The semiconductor industry experiences rapid technological advancements and hazardous materials used in the process change frequently [8]. Risk factors for reproductive toxicity in the semiconductor industry include physical factors, such as exposure to ionizing and non-ionizing radiation; ergonomic factors, such as heavy lifting, a work posture that requires standing for long durations; shift work; and work-related stress [9]. Moreover, interactions between these factors might occur. Therefore, when conducting an exposure assessment in the semiconductor industry, it is difficult to limit the exposure to specific risk factors; thus, an assessment of occupational exposure in the semiconductor industry should include production periods [7].

Previous studies on factors that affect the health of workers in the semiconductor industry focused on cancers and reproductive toxicity [10]. Reproductive toxicity (including spontaneous abortion, infertility, menstrual irregularities, low birth weight, preterm delivery, and congenital anomalies) affects women of childbearing age who are exposed to occupational hazards from production work. Spontaneous abortion can reveal occupational risks more quickly than post-delivery pregnancy outcomes, such as congenital anomalies or stillbirth; thus, it is an important criterion for examining reproductive toxicity in the semiconductor industry [11]. A study of female workers in the Korean electronics industries showed that they had a higher risk of spontaneous abortion than the control groups (women who were not exposed to occupational hazards, such as economically inactive women, the female working population as a whole, and female workers employed in the banking industry) [12]. This was the first study to examine reproductive toxicity in female workers who produced semiconductors and circuit boards in the Korean electronics industries. However, because this study examined female workers in the electronics industry, reproductive toxicity in female semiconductor workers could not be accurately assessed. Furthermore, the study used claims data from the National Health Insurance rather than data collected for the study objectives.

Reproductive toxicity in female semiconductor workers is an important issue that has received increasing attention. Thus, our study aimed to investigate the risk for spontaneous abortion in female semiconductor workers to assess reproductive toxicity in the semiconductor industry. We analyzed differences in the risk for spontaneous abortion by job type and year of pregnancy.

## Methods

### Study population

**Fig. 1:**
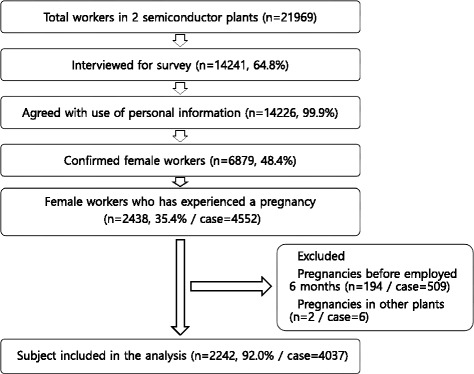
Selection of study population

A structured questionnaire survey was administered to current female workers of two semiconductor manufacturing plants (A and B plants) by a trained surveyor between April 9 and May 21, 2015. Prior to the survey, researchers explained the objectives and methods of the study. Of 21,969 employees registered in the personnel records, 14,241 (64.8%) participated in the survey and 14,226 (99.9%) consented to the use of the survey data. Of the 6879 female workers who participated in the survey, 2438 women experienced 4552 pregnancies. We included female workers currently working in the semiconductor manufacturing industry who experienced a pregnancy at least 6 months after the start of their employment with the company. We excluded cases in which the timing or outcome of the pregnancy (e.g., nulligravida, current pregnancy) could not be confirmed and cases in which the pregnancy occurred while working somewhere other than the two plants included in this study. Ultimately, 2242 female workers and 4037 pregnancies were analyzed (Fig. 1).

This study was approved by the Institutional Review Board of the Korea National Open University (ABN01–201502–11-02). Informed consent was received from the subjects prior to administering the questionnaire survey.

### Exposure assessment

Since June 1996, the semiconductor company in this study has electronically recorded personnel data. We classified exposure based on job information contained in these personnel records. Subjects were classified into 18 groups according to their job characteristics based on professional guidance from the company’s department of environmental safety. After determining the intensity and duration of exposure to harmful factors for each group, we estimated the potential risk according to job type. We then categorized the subjects into three groups based on the characteristics of exposure as follows.

First, personnel records were used to divide the subjects into clerical workers (who worked weekdays in the office) and production workers (who worked shifts on the production floor). The production workers were further divided into fabrication (FAB) and packaging (PKG) process workers. Most of the female production workers were operators. To increase the specificity of the workers’ exposure, field engineers and office-based engineers were included in the group of clerical workers.

The semiconductor industry tends to hire workers who are capable of performing specific work and are likely to remain in their jobs. However, some workers may be required to perform more than one job during their employment. In these instances, we determined which position was held the longest, which is the most common method for job classification and best represents the worker’s occupational experience [13]. The last job and the job held longest were the same for all pregnancy cases, and the concordance rate for the first job and the job held longest was 91.5%.

For an assessment of reproductive toxicity, it is important to determine the time window of exposure. Short-term exposure before pregnancy is critical, but long-term exposure can also cause irreversible changes in germ cells [14]. However, it is difficult to independently evaluate the exposure to each chemical because workers are simultaneously exposed to a number of different chemicals during the semiconductor production process [7]. Accordingly, the job held longest was used as the independent variable.

### Pregnancy outcomes

We investigated pregnancy outcomes and end-points. Pregnancy outcomes were classified as full-term delivery, preterm delivery, stillbirth, spontaneous abortion, and induced abortion. Spontaneous abortion was defined as a pregnancy being terminated before 20 weeks; in these cases, the gestational age (GA) was assessed in weeks. Full-term and preterm deliveries were defined as delivery at GAs of ≥37 and <37 weeks, respectively, whereas stillbirth was defined as infant death at a GA of ≥20 weeks. Because the end-points of the pregnancies were surveyed in date format (year and month), pregnancy duration was converted to months to estimate the start of pregnancy. Consequently, the GA of spontaneous abortion cases was converted to months. The GAs of full-term delivery, preterm delivery, stillbirth, and induced abortion were assumed to be 10, 9, 5, and 3 months, respectively.

The questionnaire was designed by the researchers for the purpose of this study. The questionnaire included information on demographic characteristics, environmental and occupational characteristics, medical history, mental health, safety and health systems, compensation, reproductive toxicity, and women’s health. Data on the women’s demographic characteristics (age at conception, body mass index [BMI] at interview, educational level at interview, and smoking status at interview) and occupational characteristics (duration of employment at conception, year of conception, study plant employed at conception, and work schedule at conception) were used for analysis in the survey. Smoking status was categorized as current/former or never smokers. Pregnancy-related survey data were used to derive the obstetric history. Gravidity was calculated as the number of pregnancies, including the current pregnancy. The obstetric history was classified according to pregnancy outcome prior to pregnancy, including full-term delivery, preterm delivery, spontaneous abortion, induced abortion, and stillbirth.

### Statistical analysis

Demographic and occupational characteristics as well as obstetric history were compared by job type. To compare ages at conception as continuous variables, a Welch’s analysis of variance (ANOVA) was performed. Because the duration of employment did not show a normal distribution, a Kruskal-Wallis test was conducted. For all other categorical variables, the chi-square test was used.

A logistic regression analysis was performed to assess the risk of spontaneous abortion in female semiconductor workers according to their job type. Model 1 was adjusted for the confounding variables of age at conception, BMI, educational level, and smoking status; model 2 was adjusted for all variables in Model 1 plus pregnancy outcome prior to pregnancy; and model 3 was adjusted for all variables in Model 2 plus duration of employment at pregnancy, year of conception, work location, and shift work.

Two different methods were used to compare the risks for spontaneous abortion associated with the occupational exposure of female semiconductor workers. First, the odds ratios (OR) of spontaneous abortion in relation to the job held longest were estimated using log-binomial regression. Generalized estimating equations (GEEs) with exchangeable working correlation structures were used to account for within-person correlations between pregnancies [15]. Second, logistic regression analysis was limited to the first pregnancies of the female workers after they joined the company to satisfy the assumption of independence among pregnancies [16, 17]. Moreover, we stratified the analysis by time period of conception (prior to 2008 vs. after 2009) to analyze the differences in exposure based on semiconductor production periods. To select subjects who had no job changes after joining the company for the sensitivity analysis, the analysis was limited to women whose first job, last job, and job held longest were identical.

SPSS version 23 (IBM Corporation, Armonk, NY, USA) was used for statistical analysis. ORs and 95% confidence intervals (CIs) are presented. A *p*-value of <0.05 in a two-tailed test was considered to be statistically significant.

## Results

The mean age during pregnancy was lower for FAB workers (27.7 ± 3.5 years) and PKG workers (27.8 ± 3.5 years) than for clerical workers (29.6 ± 3.1 years). FAB and PKG workers were more likely to be obese (BMI ≥25 kg/m^2^), have lower educational levels, and be a current or former smokers than were clerical workers. PKG workers had longer durations of employment at conception than FAB and clerical workers. The A plant had a higher percentage of clerical workers than the B plant. Regarding the women’s work schedules, FAB and PKG workers mostly worked rotating shifts, whereas clerical workers mostly worked fixed daytime hours. We found no significant differences in pregnancy outcomes prior to pregnancy or year of conception between the three types of workers (Table 1).

**Table 1:** Characteristics of 4037 pregnancies of female workers in the semiconductor industry

	The job held longest				
	FAB (*N* = 2314)	PKG (*N* = 1372)	Clerical worker (*N* = 351)	Total (*N* = 4037)	*P*-value
Age at conception (years)					
Mean ± SD	27.7 ± 3.5	27.8 ± 3.5	29.6 ± 3.1	27.8 ± 3.5	<0.001^a^
Post hoc test^b^	b	b	a		
≤29	1747 (75.5)	1023 (74.6)	195 (55.6)	2965 (73.4)	<0.001
30–34	506 (21.9)	304 (22.2)	141 (40.2)	951 (23.6)	
≥35	61 (2.6)	45 (3.3)	15 (4.3)	121 (3.0)	
BMI at interview (kg/m^2^)					
≤24.9	1911 (82.6)	1104 (80.5)	320 (91.2)	3335 (82.6)	<0.001
≥25.0	403 (17.4)	268 (19.5)	31 (8.8)	702 (17.4)	
Level of education at interview					
High school	1521 (65.7)	1000 (72.9)	81 (23.1)	2602 (64.5)	<0.001
College/university	793 (34.3)	372 (27.1)	270 (76.9)	1435 (35.5)	
Smoking status at interview					
Non smoker	1843 (79.6)	1114 (81.2)	341 (97.2)	3298 (81.7)	<0.001
Smoker	471 (20.4)	258 (18.8)	10 (2.8)	739 (18.3)	
Pregnancy outcome prior to pregnancy					
Primigravida	1232 (53.2)	744 (54.2)	180 (51.3)	2156 (53.4)	0.358
Full term delivery	784 (33.9)	426 (31.0)	124 (35.3)	1334 (33.0)	
Preterm delivery	32 (1.4)	18 (1.3)	7 (2.0)	57 (1.4)	
Spontaneous abortion	200 (8.6)	137 (10.0)	24 (6.8)	361 (8.9)	
Induced abortion	52 (2.2)	38 (2.8)	13 (3.7)	103 (2.6)	
Stillbirth	14 (0.6)	9 (0.7)	3 (0.9)	26 (0.6)	
Duration of employment at conception (years)					
Median (IQR)	7.6 (5.3–10.5)	8.0 (5.6–10.9)	7.3 (4.9–10.5)	7.8 (5.3–10.7)	0.009^c^
Post hoc test^d^	a	b	a		
≤4	523 (22.6)	255 (18.6)	94 (26.8)	872 (21.6)	0.004
5–9	1123 (48.5)	685 (49.9)	154 (43.9)	1962 (48.6)	
≥10	668 (28.9)	432 (31.5)	103 (29.3)	1203 (29.8)	
Year of conception					
≥2009	1466 (63.4)	867 (63.2)	212 (60.4)	2545 (63.0)	0.559
≤2008	848 (36.6)	505 (36.8)	139 (39.6)	1492 (37.0)	
Study plant					
A plant	1369 (59.2)	740 (53.9)	274 (78.1)	2383 (59.0)	<0.001
B plant	945 (40.8)	632 (46.1)	77 (21.9)	1654 (41.0)	
Work schedule					
Days only	235 (10.2)	125 (9.1)	299 (85.2)	659 (16.3)	<0.001
Rotating shift	2079 (89.8)	1247 (90.9)	52 (14.8)	3378 (83.7)	

FAB fabrication process worker, PKG package process worker

Smokers included current and former smokers

*SD* standard deviation, *BMI* body mass index, *IQR* interquartile range

^a^Welch one-way analysis of variance

^b^Games-Howell test

^c^Kruskal-Wallis test

^d^Pairwise comparisons

In the regression analysis, the job type and risk for spontaneous abortion were not significantly different in the unadjusted and adjusted regression models. However, age at conception of ≥30 years, a history of smoking, and spontaneous abortion prior to pregnancy were associated with higher odds for spontaneous abortion (Table 2).

**Table 2:** Crude and adjusted odds ratios of spontaneous abortion by risk factors of female workers employed in the semiconductor industry

	Pregnancy	SA	Crude		Adjusted					
					Model 1^a^		Model 2^b^		Model 3^c^	
	(N = 4037)	(*N* = 529)	OR	(95% CI)	OR	(95% CI)	OR	(95% CI)	OR	(95% CI)
The job held longest										
FAB	2314	291 (12.6)	1.15	0.81–1.64	1.09	0.71–1.67	1.08	0.70–1.66	1.08	0.64–1.81
PKG	1372	199 (14.5)	1.36	0.94–1.96	1.28	0.82–1.99	1.26	0.81–1.97	1.24	0.72–2.12
Clerical worker	351	39 (11.1)	1.0		1.0		1.0		1.0	
Age at conception (years)										
≤29	2965	354 (11.9)	1.0		1.0		1.0		1.0	
30–34	951	141 (14.8)	**1.28**	**1.04–1.58**	**1.35**	**1.08–1.69**	**1.34**	**1.06–1.70**	**1.34**	**1.01–1.78**
≥35	121	34 (28.1)	**2.88**	**1.91–4.35**	**2.98**	**1.94–4.59**	**2.93**	**1.88–4.55**	**2.95**	**1.79–4.85**
BMI at interview (kg/m^2^)										
≤24.9	3335	421 (12.6)	1.0		1.0		1.0		1.0	
≥25.0	702	108 (15.4)	**1.26**	**1.00–1.58**	1.16	0.91–1.48	1.15	0.90–1.48	1.16	0.91–1.48
Level of education at interview										
High school	1435	185 (12.9)	1.0		1.0		1.0		1.0	
College/university	2602	344 (13.2)	1.03	0.85–1.25	1.04	0.84–1.29	1.03	0.84–1.28	1.08	0.87–1.34
Smoking status at interview										
Non smoker	3298	410 (12.4)	1.0		1.0		1.0		1.0	
Smoker	739	119 (16.1)	**1.35**	**1.08–1.69**	**1.44**	**1.14–1.81**	**1.42**	**1.13–1.80**	**1.37**	**1.08–1.73**
Pregnancy outcome prior to pregnancy										
Primigravida	2156	267 (12.4)	1.0				1.0		1.0	
Full term delivery	1334	166 (12.4)	1.01	0.82–1.24			0.95	0.76–1.20	0.92	0.73–1.16
Preterm delivery	57	6 (10.5)	0.83	0.35–1.96			0.87	0.36–2.09	0.84	0.35–2.01
Spontaneous abortion	361	69 (19.1)	**1.67**	**1.25–2.24**			**1.58**	**1.16–2.15**	**1.53**	**1.12–2.08**
Induced abortion	103	15 (14.6)	1.21	0.69–2.12			0.79	0.40–1.56	0.81	0.41–1.59
Stillbirth	26	6 (23.1)	2.12	0.84–5.33			1.63	0.59–4.49	1.70	0.62–4.70
Duration of employment at conception (years)										
≤4	872	93 (10.7)	1.0						1.0	
5–9	1962	268 (13.7)	**1.33**	**1.03–1.70**					1.26	0.97–1.65
≥10	1203	168 (14.0)	**1.36**	**1.04–1.78**					1.08	0.76–1.52
Year of conception										
≥2009	2545	372 (14.6)	1.0						1.0	
≤2008	1492	157 (10.5)	**0.69**	**0.56–0.84**					0.80	0.64–1.00
Study plant										
A plant	2383	313 (13.1)	1.0						1.0	
B plant	1654	216 (13.1)	0.99	0.82–1.20					1.01	0.82–1.23
Work schedule										
Days only	659	82 (12.4)	1.0						1.0	
Rotating shift	3378	447 (13.2)	1.07	0.83–1.38					0.97	0.69–1.38

FAB fabrication process worker, PKG package process worker

Smokers included current and former smokers

*SA* spontaneous abortion, *OR* odds ratio, *CI* confidence interval

Values displaying significant differences are shown in bold (*p*-value ≤ 0.05)

^a^Multiple logistic regression model adjusted for the job held longest, age, BMI, education, smoking

^b^Multiple logistic regression model adjusted for the job held longest, age, BMI, education, smoking, pregnancy outcome just prior to pregnancy

^c^Multiple logistic regression model adjusted for the job held longest, age, BMI, education, smoking, pregnancy outcome prior to pregnancy, duration of employment, year of conception, study plant, shift work

When analyses were performed using a GEE and limited to first pregnancies after joining the company, the risks for spontaneous abortion were not significantly different among job types. When the analysis was limited to first pregnancies after joining the company, the OR for spontaneous abortion was higher than that in the GEE analysis (Tables 3 and 4).

**Table 3:** Odds ratios of spontaneous abortion among generalised estimate equation of all pregnancies

	Pregnancy	SA	Crude GEE		Adjusted GEE^a^	
	(N = 4037)	(N = 529)	OR	(95% CI)	OR	(95% CI)
The job held longest						
FAB	2314	291 (12.6)	1.12	0.81–1.57	1.12	0.79–1.58
PKG	1372	199 (14.5)	1.28	0.91–1.81	1.26	0.88–1.79
Clerical worker	351	39 (11.1)	1.0		1.0	
Age at conception (years)						
≤29	2965	354 (11.9)	1.0		1.0	
30–34	951	141 (14.8)	**1.25**	**1.03–1.50**	**1.28**	**1.02–1.60**
≥35	121	34 (28.1)	**2.41**	**1.75–3.33**	**2.48**	**1.69–3.64**
Duration of employment (years)						
≤4	872	93 (10.7)	1.0		1.0	
5–9	1962	268 (13.7)	**1.29**	**1.02–1.63**	1.22	0.96–1.54
≥10	1203	168 (14.0)	**1.37**	**1.06–1.78**	1.06	0.78–1.43
Year of conception						
≥2009	2545	372 (14.6)	1.0		1.0	
≤2008	1492	157 (10.5)	**0.73**	**0.60–0.88**	0.84	0.68–1.02

FAB fabrication process worker, PKG package process worker

*GEE* generalized estimating equation, *OR* odds ratio, *CI* confidence interval

Values displaying significant differences are shown in bold (*p*-value ≤ 0.05)

^a^Adjusted for age, BMI, education, smoking, duration of employment, year of conception, study plant

**Table 4:** Odds ratios of spontaneous abortion among logistic regression model of first pregnancies

	Pregnancy	SA	Crude logistic		Adjusted logistic^a^	
	(*N* = 2242)	(*N* = 98)	OR	(95% CI)	OR	(95% CI)
The job held longest						
FAB	1285	54 (4.2)	1.40	0.60–3.31	1.15	0.46–2.85
PKG	759	38 (5.0)	1.69	0.70–4.05	1.34	0.52–3.41
Clerical worker	198	6 (3.0)	1.0		1.0	
Age at conception (years)						
≤29	1886	71 (3.8)	1.0		1.0	
30–34	326	21 (6.4)	**1.76**	**1.07–2.91**	1.31	0.70–2.47
≥35	30	6 (20.0)	**6.39**	**2.53–16.12**	**4.18**	**1.40–12.43**
Duration of employment (years)						
≤4	664	24 (3.6)	1.0		1.0	
5–9	1167	48 (4.1)	1.14	0.69–1.89	0.95	0.56–1.58
≥10	411	26 (6.3)	**1.80**	**1.02–3.18**	1.30	0.63–2.67
Year of conception						
≥2009	1275	82 (6.4)	1.0		1.0	
≤2008	967	16 (1.7)	**0.24**	**0.14–0.42**	**0.32**	**0.18–0.55**

FAB fabrication process worker, PKG package process worker

*OR* odds ratio, *CI* confidence interval

Values displaying significant differences are shown in bold (*p*-value ≤ 0.05)

^a^Adjusted for age, BMI, education, smoking, duration of employment, year of conception, study plant

Among women who conceived prior to 2008, the risk of spontaneous abortion was significantly higher for PKG workers than for clerical workers (OR 2.21, 95% CI: 1.01–4.81). In contrast, among women who conceived after 2009, there were no differences in the risk for spontaneous abortion according to job type (Table 5). The association between job type and the risk for spontaneous abortion was also analyzed in a sensitivity analysis that was limited to women whose first job, last job, and job held longest were identical; no significant differences in the OR for spontaneous abortion by job type were found in this analysis (Table 6).

**Table 5:** Odds ratios of spontaneous abortion among generalised estimate equation of all pregnancies stratified by year of conception

Year of conception	The job held longest	Pregnancy	SA	Crude GEE		Adjusted GEE^a^	
		(*N* = 4037)	(*N* = 529)	OR	(95% CI)	OR	(95% CI)
≤2008	FAB	848	80 (9.4)	1.62	0.75–3.49	1.54	0.71–3.32
	PKG	505	69 (13.7)	**2.31**	**1.06–5.02**	**2.21**	**1.01–4.81**
	Clerical worker	139	8 (5.8)	1.0		1.0	
≥2009	FAB	1466	211 (14.4)	0.98	0.68–1.40	1.01	0.69–1.48
	PKG	867	130 (15.0)	1.03	0.71–1.49	1.05	0.71–1.55
	Clerical worker	212	31 (14.6)	1.0		1.0	

FAB fabrication process worker, PKG package process worker

*GEE* generalized estimating equation, *SA* spontaneous abortion, *OR* odds ratio, *CI* confidence interval

Values displaying significant differences are shown in bold (*p*-value ≤ 0.05)

^a^Adjusted for age, BMI, education, smoking, duration of employment, study plant

**Table 6:** Odds ratios of spontaneous abortion among generalized estimate equation of all pregnancies, limited to female workers whose first, last, and job held longest were the same

Category	The job matching	Pregnancy	SA	Crude GEE		Adjusted GEE^a^	
		(*N* = 3692)	(*N* = 489)	OR	(95% CI)	OR	(95% CI)
3 category	FAB	2244	285 (12.7)	1.12	0.76–1.64	1.07	0.71–1.60
	PKG	1193	175 (14.7)	1.28	0.86–1.91	1.19	0.79–1.81
	Clerical worker	255	29 (11.4)	1.0		1.0	
2 category	Process worker^b^	3437	460 (13.4)	1.18	0.81–1.72	1.11	0.75–1.65
	Clerical worker	255	29 (11.4)	1.0		1.0	

FAB fabrication process worker, PKG package process worker

*GEE* generalized estimating equation, *SA* spontaneous abortion, *OR* odds ratio, *CI* confidence interval

^a^Adjusted for age, BMI, education, smoking, duration of employment, year of conception, study plant

^b^Process worker included fabrication process worker and package process worker

## Discussion

This study investigated the association between the risk for spontaneous abortions and the job type of female semiconductor workers to assess their reproductive health. The risk for spontaneous abortion for female semiconductor workers was not significantly higher for FAB and PKG workers than for clerical workers according to their job held longest. However, when the analysis was stratified for the year of conception, we found significantly higher odds for spontaneous abortions (OR 2.21, 95% CI: 1.01–4.81) in PKG workers than clerical workers when the pregnancy occurred prior to 2008.

In this study, the spontaneous abortion rates for FAB and PKG workers were 12.6% and 14.5%, respectively; these were higher than the rate of 11.1% found in clerical workers. These spontaneous abortion rates for female workers involved in semiconductor production were higher than the spontaneous abortion rate of 11.1% reported for Korean domestic 15- to 44-year-old married women [18]. In 1988, Pastides et al. first reported data on reproductive toxicity in the semiconductor industry using a questionnaire survey. They showed that the risk for spontaneous abortion was higher for diffusion and photolithographic process workers than for non-FAB workers [19]. Later, the University of California conducted two cohort studies with support from the Semiconductor Industry Association in the United States. The first study—a retrospective cohort study using questionnaire data and medical records—showed that FAB workers had higher odds for experiencing a spontaneous abortion than did non-FAB workers (OR 1.43, 95% CI: 0.95–2.09). Women working in masking and etching-related processes, in particular, had a significantly higher risk for spontaneous abortion [20]. In the second study (a prospective cohort study using urine samples that were collected daily for 6 months), FAB workers again had a higher risk for spontaneous abortion than did non-FAB workers (OR 1.25, 95% CI: 0.63–1.76) [21]. An epidemiological study on reproductive toxicity in the semiconductor industry revealed that the risk for spontaneous abortions tended to be higher for FAB workers; in particular, when the analysis was broken down by sub-processes, job types, and level of exposure to hazards, the risk for reproductive toxicity was significantly increased [7].

Other studies reported that the work processes of the semiconductor industry were not associated with an increased risk for spontaneous abortions. In a case-control study including patients diagnosed with intrauterine spontaneous abortions based on pathologic results, FAB workers did not have an increased risk for spontaneous abortions when compared with non-FAB workers (OR 0.87, 95% CI: 0.45–1.60) [22]. A nested case-control study on current and former female semiconductor workers showed an increased risk for spontaneous abortions in FAB workers (OR 0.58, 95% CI: 0.26–1.30) compared with non-FAB workers [23]. These conflicting results might be caused by the relatively low numbers of pregnancies among the FAB workers in the studies (15 and 36, respectively).

In previous studies, exposure classification of the subjects consisted mainly of FAB versus non-FAB workers or different FAB sub-processes or hazards [7]. Workers may be exposed to a number of substances associated with reproductive toxicity during the FAB process in the semiconductor industry, including carbon monoxide, N,N-dimethylacetamide, 2-ethoxyethanol, ethylbenzene, ethylene glycol, 2-methoxy-1-propanol, 2-methoxy-1-propylacetate, nitrous oxide, and xylene. During the PKG process, workers may be exposed to antimony trioxide, N,N-dimethylformamide, ethylbenzene, ethylene oxide, methanol, phenol, trichloroethylene, and xylene (Additional file 1: Table S1) [24]. Previous studies have shown that ethylene glycol ether, isopropyl alcohol, xylene, and butyl acetate are significantly associated with an increased risk for spontaneous abortions in FAB workers. Among the FAB sub-processes, photolithography, diffusion, masking, dopant application, thin film application, and etching are known to increase the risk for spontaneous abortions [7].

The PKG process consists of wafer dicing, where the wafer is cut into chips; die bonding, where the chips are connected to the circuit board; wire bonding, where the chips are connected to the circuit board with a gold wire; molding, where the chips are wrapped with resin; and testing, where electrical and thermal stress are applied to the chips. During molding, which is performed to protect the semiconductor chips from the environment, an epoxy molding compound (EMC) is used under high-temperature conditions (180 °C). Although the EMC is a high-molecular-weight polymer, volatile organic compounds (VOCs; in particular, benzene and methylisobutylketone) and formaldehyde can be generated under heat. In the FAB and PKG processes, general ventilation is implemented based on particle number concentration, but mixing of indoor air occurs between the sub-processes. Therefore, VOCs and formaldehyde that are PKG-generated by-products can affect other nearby sub-processes [6]. Moreover, the testing process can include ionizing radiation exposure. Because semiconductor equipment is densely arranged, continued exposure to extremely low frequency magnetic fields (ELF-MF) may occur [25]. In a previous study on the association between exposure to petrochemicals as an occupational hazard and the risk for spontaneous abortions, the risk for spontaneous abortion was significantly increased by exposure to benzene, gasoline, and hydrogen sulfide [26]. In a systematic review using a meta-analysis, women who were exposed to formaldehyde showed a significantly increased risk for spontaneous abortions [27]. Future studies on the relationship between reproductive toxicity and exposure to VOCs, formaldehyde, ionizing radiation, and ELF-MF in the semiconductor industry are needed.

The weight of the wafer increases along with its diameter, which makes manual handling of wafers difficult. To overcome this issue, many processes have become automated and older processes, chemicals, and operational methods that had been used to produce smaller wafers are being phased out [28, 29]. Therefore, differences in occupational exposure based on the semiconductor production period must be considered. In the late 2000s, semiconductor production infrastructure changed from a wafer diameter of 8 in. to one of 12 in. [30]. It is likely that the level of exposure to occupational hazards differed depending on the semiconductor production period; consequently, the risk for spontaneous abortions would be higher during the PKG process used in the past. In this study, when female semiconductor workers became pregnant prior to 2008, the risk for spontaneous abortions in PKG workers was significantly higher than that in clerical workers. When female workers who entered the company before 2008 experienced repetitive pregnancies, the age and gravidity of pregnancy prior to 2008 was lower than those for pregnancies after 2009. This may result in an increase in the average age of pregnancy after 2009 (Additional file 1: Table S2). The average age of the first pregnancy for women who entered the company after 2009 was also higher than for women who began their employment prior to 2008 (Additional file 1: Table S3). Thus, the age at which female workers begin to experience a pregnancy is now higher than in the past. Therefore, pregnancies that occurred after 2009 are more likely to be at high risk for spontaneous abortions.

There is a critical period of reproductive toxicity determined by occupational hazards. Reproductive toxicity can manifest as various features depending on which hazard the worker was exposed to and when. Therefore, it is critical to identify an adequate exposure assessment period for determining reproductive toxicity in women. Maternal exposure in the few months before conception is important for infertility, and teratogenic effects may arise during the organogenesis phase during the first 3–8 weeks of pregnancy. Long-term exposure may cause irreversible changes to the DNA of germ cells, and certain substances may persist and concentrate in body fat [14]. Reproductive toxicity can be the result of chronic or acute exposure. In the assessment of the cumulative effects of hazards, we found no differences in the risk for spontaneous abortions according to the duration of employment. In this study, the time window of occupational exposure was assumed to be 6 months or longer [17, 31].

When comparing the first job and the job held longest, we found few cases in which the employee changed from a clerical worker to a production worker (Additional file 1: Table S4). When the classification of exposure was selected as the job held longest, there may be misclassification bias due to the inclusion of some production workers as clerical workers, which might underestimate the risk of spontaneous abortion for PKG workers. The median duration of employment at pregnancy was 7.8 years (interquartile range: 5.3–10.7). Female workers were likely to become pregnant after working at the company for some time. Therefore, this study classified the exposure according to the job held longest.

The risk factors for spontaneous abortion have been shown to include the mother’s age of ≥35 years, a history of spontaneous abortions, gravidity, smoking, BMI, and educational level [32–37]. In this study, the risk for spontaneous abortion was significantly higher for mothers who were ≥35 years old, had a history of spontaneous abortions, and were current and former smokers. However, BMI and educational level were not significantly associated with the risk for spontaneous abortions. In this study, BMI and educational level were assessed at the time of the survey and not during pregnancy. Therefore, misclassification bias might have affected the results.

The limitations in studying the health impact of working in the semiconductor industry include the healthy worker and infertile worker effects [7, 12, 23]. The *healthy worker effect* refers to the concept that healthy people have a higher probability of being hired and of remaining at their workplace, whereas those who are not healthy are more likely to quit their jobs. The *infertile worker effect* refers to the phenomenon of infertile women or those without children remaining at work or female workers leaving their jobs during their pregnancy. These effects can be compensated for by selecting an external control group with similar forms of employment or an internal control group with a lower occupational exposure level, rather than selecting from the general population. In this study, the healthy and infertile worker effects were minimized by adjusting for age at the time of pregnancy and work period; moreover, we selected clerical workers who were employed in the semiconductor industry as the control group [38].

When studying reproductive toxicity in semiconductor processes, it is necessary to define pregnancy. This study confirmed pregnancies and pregnancy outcomes based on data from the questionnaire survey. Because a spontaneous abortion is a traumatic event, the subjects might have found it difficult to respond to questions about spontaneous abortion. The possibility of such a recall bias occurring in only one group among the production and clerical worker groups is very low; this can underestimate the effects of occupational hazards on the risk for spontaneous abortions by non-differential misclassification bias [23].

A surveillance system of reproductive health is needed to assess and monitor the risks, working conditions, and hazardous materials to which future childbearing-age female workers may be exposed. Moreover, individuals who were employed during periods when working conditions were poor and not managed properly should be followed up to further assess reproductive toxicity according to semiconductor production periods.

## Conclusions

In this study, we administered a reproductive health-related questionnaire survey to current female workers in the semiconductor industry. We found that female PKG workers who worked in the semiconductor industry prior to 2008 had an increased risk for spontaneous abortions. In previous studies investigating reproductive toxicity, the focus was placed on FAB and its sub-processes; however, our data indicate that an assessment of reproductive toxicity associated with PKG processes is needed. These results could be used as a basis for future maternity protection policies.

## Additional file

Additional file 1: Table S1. Substances causing reproductive toxicity in semiconductor industry. **Table S2.** Characteristics of 4037 all pregnancies of female workers in the semiconductor industry. **Table S3.** Characteristics of 2242 first pregnancies of female workers in the semiconductor industry. **Table S4.** Comparison of the first job and the job held longest. (XLSX 31 kb)

## Abbreviations

ANOVA: Analysis of variance
CI: Confidence interval
ELF-MF: Low-frequency magnetic field
EMC: Epoxy molding compound
FAB: Fabrication
GA: Gestational age
GEE: Generalized estimating equation
KWCWS: Korea workers’ compensation and welfare service
OR: Odds ratio
PKG: Packaging
VOC: Volatile organic compound
